# Visual Instance-aware Prompt Tuning

**DOI:** 10.1145/3746027.3754858

**Published:** 2025-10-27

**Authors:** Xi Xiao, Yunbei Zhang, Xingjian Li, Tianyang Wang, Xiao Wang, Yuxiang Wei, Jihun Hamm, Min Xu

**Affiliations:** University of Alabama at Birmingham, Birmingham, United States; Tulane University, New Orleans, United States; Carnegie Mellon University, Pittsburgh, United States; University of Alabama at Birmingham, Birmingham, United States; Oak Ridge National Laboratory, Oak Ridge, United States; Georgia Institute of Technology, Atlanta, United States; Tulane University, New Orleans, United States; Carnegie Mellon University, Pittsburgh, United States

**Keywords:** Prompt tuning, Vision transformer, Instance aware, Image analysis

## Abstract

Visual Prompt Tuning (VPT) has emerged as a parameter-efficient fine-tuning paradigm for vision transformers, with conventional approaches utilizing dataset-level prompts that remain the same across all input instances. We observe that this strategy results in sub-optimal performance due to high variance in downstream datasets. To address this challenge, we propose Visual Instance-aware Prompt Tuning (ViaPT), which generates instance-aware prompts based on each individual input and fuses them with dataset-level prompts, leveraging Principal Component Analysis (PCA) to retain important prompting information. Moreover, we reveal that VPT-Deep and VPT-Shallow represent two corner cases based on a conceptual understanding, in which they fail to effectively capture instance-specific information, while random dimension reduction on prompts only yields performance between the two extremes. Instead, ViaPT overcomes these limitations by balancing dataset-level and instance-level knowledge, while reducing the amount of learnable parameters compared to VPT-Deep. Extensive experiments across 34 diverse datasets demonstrate that our method consistently outperforms state-of-the-art baselines, establishing a new paradigm for analyzing and optimizing visual prompts for vision transformers.

## 1 Introduction

Large-scale vision transformers (ViTs) [16] pretrained on millions of images have become the de-facto backbone for a wide spectrum of visual recognition tasks. Visual Prompt Tuning (VPT) [3, 29] has emerged as a parameter-efficient approach for adapting these models to downstream tasks. Instead of updating the backbone through full/partial fine-tuning, a small set of learnable tokens, namely *prompts*, is prepended to the input sequence and optimized while the backbone parameters remain frozen. This strategy has been proven successful in various visual recognition scenarios.

Despite promising, existing VPT methods [3, 13, 18, 26, 29, 30, 44, 68, 72] suffer from a fundamental limitation: the prompt tokens are tied to the entire dataset that is used for tuning the backbone. Such dataset-level prompts implicitly assume that a universal set of prompts can adapt the pre-trained backbone to downstream data by reconciling the diverse semantics and visual statistics, overlooking the prompting effect that can be potentially offered by an individual input image (i.e., instance). Moreover, this assumption rarely holds in practice. For example, fine-grained recognition or domain shifts inevitably introduce substantial intra-class variability that cannot be effectively captured by a single set of prompts. Fig. 1 illustrates this phenomenon on the CUB-200 dataset: the performance of VPT-Shallow/Deep could be further boosted with instance-aware prompts (pink vs blue). Similarly, class imbalance also undermines this assumption, partially explaining why typical VPT methods [18, 29, 44, 72, 73] overfit the dominant classes in a dataset. These observations suggest that a single set of dataset-level prompts is inherently limited and that instance sensitivity should be encouraged for robust adaptation.

We address this limitation by proposing Visual Instance-aware Prompt Tuning (**ViaPT**). To exploit the prompting effect of an instance, the first step is to determine how to involve the image information in the prompt. A straightforward option could be using a lightweight learnable encoder to extract image features and use them directly as instance-aware prompts. However, we observe that this fashion yields sub-optimal performance, and incurs more learnable parameters. Therefore, we are motivated to design a new fashion. Specifically, we still use a lightweight encoder that accepts the image as input, aiming to learn the statistics (i.e., mean and std) of the distribution, from which the image comes. Afterwards, we randomly sample variables from N(0,I) and then generate instance-aware prompts by scaling and shifting the sampled variables based on the learned mean and std [36]. To avoid missing global information, we simultaneously use dataset-level prompts along with the proposed instance-aware ones. This is achieved by firstly combining them with regular concatenation, and then applying PCA to the concatenated prompts to retain the most important prompting information. Since VPT requires the same dimension for both image and prompt tokens, we pad the prompts, of which dimension is reduced by PCA, with randomized and learnable parameters, which also increase the dynamics of the prompts. The generation of instance-aware prompts and the use of PCA are integrated into an end-to-end learning pipeline, which has fewer parameters than VPT-Deep and most of its variants (see Table 1). Notably, a certain level of randomness is involved in fine-tuning because of the sampling operations used to generate instance-aware prompts, however, to avoid such randomness during inference, we only sample needed variables once from N(0,I) beforehand with a fixed seed for all testing images, thus the instance-aware prompts are mainly determined by the *mean* and *std* associated with a testing image.

Furthermore, in terms of the prompting fashions between the transformer layers in a model, we find that the proposed method along with the typical VPT schemes (i.e., VPT-Deep/Shallow) can be jointly interpreted based on a conceptual understanding. Specifically, VPT-Deep, which prepends randomized prompts to every transformer layer and jointly learns them, corresponds to setting the number of principle components to 0 in PCA (i.e., for the current layer, its prompts are *not* output by the immediate previous layer). In contrast, VPT-Shallow corresponds to setting the number of principle components to the full rank in PCA (i.e., for the current layer, its prompts are output by the immediate previous layer). Random dimension reduction, a naive alternative, falls somewhere in between, but fails to select important components of the prompts, while the proposed ViaPT yields a promising balance among these special cases, thereby achieving higher accuracy and stronger generalization. Our extensive experiments across 34 challenging benchmarks demonstrate that ViaPT consistently outperforms state-of-the-art baselines by on average while still maintaining parameter efficiency. Moreover, ViaPT is pioneering to shift the conventional dataset-level prompting to instance-level, defining a new paradigm for prompt tuning research for vision models. In addition, we also analyze the rationale of combining the instance-aware prompting and PCA in the ablation study in section 4.5. Notably, while ViaPT coherently integrates both instance-aware prompts generation and PCA, each can still be used with existing VPT schemes as a plug-and-play component. For instance, the yellow curve in Fig. 1 shows what if only PCA is applied with the conventional VPT, and the two endpoints of the blue curve yield the results of when instance-aware prompting is used with the conventional VPT without PCA.

This work makes the following contributions: (1) we develop a novel visual prompt tuning method that generates instance-aware prompts based on each individual input; (2) we propose to leverage PCA to retain the most important prompting information from both dataset-level and instance-aware prompts; (3) we introduce a conceptual understanding to interpret the proposed method ViaPT along with typical VPT schemes; (4) we demonstrate through extensive experiments on 34 diverse datasets that ViaPT establishes new state of the art while maintaining parameter efficiency.

## 2 Related Work

### 2.1 Visual Prompt Tuning

Visual Prompt Tuning (VPT) [4, 11, 29, 39, 40, 44, 47, 48, 52, 60, 66, 68] has emerged as a powerful parameter-efficient approach for adapting pre-trained Vision Transformers to downstream tasks. Inspired by prompt tuning [33] in natural language processing, VPT introduces a small set of learnable tokens that are prepended to the input sequence while keeping the backbone model frozen. [3, 29] first demonstrated VPT’s effectiveness for image classification tasks, showing competitive performance compared to full fine-tuning while updating only 0.1–1% of parameters. Multi-modal prompt tuning [17, 57, 59, 75, 77] applies this concept to joint vision-language tasks, while prompt ensembling [45] combines multiple specialized prompts for improved performance. The approach has been extended to test-time adaptation [35, 50, 61, 72, 73], where prompts are quickly optimized to handle distribution shifts without accessing training data. Despite these advances, existing VPT methods share a critical limitation: they optimize a single set of prompts at the dataset level, which remains static across all input instances. Our work addresses this limitation by introducing instance-specific prompts that dynamically adapt to individual inputs, significantly improving performance on diverse and fine-grained classification tasks.

### 2.2 Instance-Aware Adaptation in Vision Models

The importance of instance-specific adaptations has been recognized across various computer vision tasks [21, 43, 58, 60, 74, 76]. Dynamic networks [5, 21, 34, 37, 65] that adjust their computation paths based on input content have shown improved efficiency and accuracy. In the context of few-shot learning, instance-dependent prototypes [64] have demonstrated superior performance over fixed representations. More recently, instance-specific meta networks [74, 76] generate weights conditioned on input features. These approaches highlight the benefits of tailoring model behavior to individual inputs. Our work applies this insight to visual prompt tuning by generating instance-specific prompts, enabling more flexible adaptation to diverse visual patterns while maintaining parameter efficiency.

### 2.3 Information Flow in Deep Neural Networks

Understanding and optimizing information flow across network layers is crucial for deep learning performance [1, 2, 25, 28]. ResNet’s skip connections [23] and DenseNet’s dense connectivity [25] demonstrate how direct paths for information propagation can mitigate gradient problems and improve feature reuse. In transformers, techniques like layer normalization [2] and attention mechanisms facilitate information flow across tokens. More recently, there has been growing interest in selective feature propagation [1, 69], where only the most relevant information is preserved across layers. Our unified prompt framework relates to this line of research by using PCA-based dimension reduction to control information flow between transformer layers, effectively balancing between preserving valuable context and enabling new adaptations. This approach reveals connections between existing VPT variants while establishing a more flexible and effective paradigm for visual prompt tuning.

## 3 Methodology

In this section, we present our **ViaPT** (Visual Instance-aware Prompt Tuning), a framework (Fig. 2) that overcomes the limitations of static prompts in visual prompt tuning. After introducing background on conventional VPT (§3.1), we describe our instance-aware prompt generation approach (§3.2), present a unified framework for balanced prompt propagation across layers (§3.3), and discuss strategies to ensure inference stability (§3.4).

### 3.1 Preliminaries

#### Visual Prompt Tuning (VPT).

Vision Transformers (ViT) process input images by splitting them into k patches, which are subsequently transformed into image tokens $E_{0}\in R^{k\times d}$, where d is the embedding dimension. These image tokens, along with a class token $x_{0}$ (often denoted as [CLS]), are processed through N transformer layers ${\left\{L_{i}\right\}}_{i=1}^{N}$ to produce the final representation. Visual Prompt Tuning adapts pre-trained ViTs to downstream tasks by introducing a set of p learnable prompt tokens $P\in R^{p\times d}$ while keeping the backbone frozen.

Two primary variants exist: VPT-Shallow and VPT-Deep. In VPT-Shallow, prompts are inserted only at the first transformer layer $\left(L_{1}\right)$, resulting in

(1) $$\left[x_{1},Z_{1},E_{1}\right]=L_{1}\left(\left[x_{0},P_{0},E_{0}\right]\right),$$

(2) $$\left[x_{i},Z_{i},E_{i}\right]=L_{i}\left(\left[x_{i-1},Z_{i-1},E_{i-1}\right]\right)\;\text{for}\;i=2,\ldots ,N,$$

(3) $$y=\text{Head}\left(x_{N}\right),$$

where $Z_{i}\in R^{p\times d}$ represents the features computed by the i-th Transformer layer and [·,·] denotes token concatenation. The colors ■ and ■ indicate trainable and frozen parameters, respectively. In contrast, VPT-Deep introduces unique prompt tokens at each transformer layer’s input space:

(4) $$\left[x_{i},Z_{i},E_{i}\right]=L_{i}\left(\left[x_{i-1},P_{i-1},E_{i-1}\right]\right)\;\text{for}\;i=1,\ldots ,N,$$

(5) $$y=\text{Head}\left(x_{N}\right),$$

where $P_{i-1}\in R^{p\times d}$ are the layer-specific learnable prompts.

These learnable parameters are optimized over the entire training set and remain fixed during inference, making them effective task-specific prompts. However, this dataset-level optimization often fails to account for instance-specific variations, leading to suboptimal performance when faced with diverse visual patterns within the same task.

### 3.2 Instance-aware Prompt Generation

To move beyond dataset-level static prompts and account for input-specific visual variations, we divide the original prompts in the first layer $P_{0}:=\left\{p_{0}^{1},p_{0}^{2},\ldots ,p_{0}^{p}\right\}$ into two sets: the first λ tokens form the instance-aware prompt $P_{0}^{\text{ins}}:=\left\{p_{0}^{1},p_{0}^{2},\ldots ,p_{0}^{\lambda }\right\}$ and the remaining p-λ tokens comprise the dataset-level prompt $P_{0}^{\text{dom}}:=\left\{p_{0}^{\lambda +1},p_{0}^{\lambda +2},\ldots ,p_{0}^{p}\right\}$. The dataset-level prompts are initialized and updated following standard VPT-Shallow/Deep approaches. For instance-aware prompts, our goal is to design a prompt generator that takes input data and produces corresponding prompt tokens.

While using raw images as input is intuitive, it introduces significant computational overhead. Instead, we leverage the image tokens $E_{0}$, which represent images in a much lower dimension to improve efficiency. We define the instance prompt generator as $g:R^{k\times d}\to R^{\lambda \times d}$, where for each input $E_{0}$, the generator synthesizes λ prompt tokens $g\left(E_{0}\right)$, which are then concatenated with the dataset-level prompts for prompt tuning.

Although the generator introduces additional trainable parameters beyond the prompts, this is necessary to capture instance-level characteristics. To maintain both efficacy and parameter efficiency, we simply use a learnable 2-layer convolutional encoder as the generator, which performs well as demonstrated in our experiments. To further reduce trainable parameters in line with PEFT principles, we find that learning a probabilistic generator is more effective than a deterministic one. This approach reduces the output of g to $R^{2\times d}$ instead of $R^{\lambda \times d}$, significantly decreasing parameter count and computation. Furthermore, this operation constrains the instance-aware prompt distribution to a Gaussian N(0,I), which aligns with the initialization of dataset-level prompts and mitigates magnitude discrepancies between the two prompt types. For each set of image tokens $E_{0}$, the generator g returns mean and standard deviation pairs: $(\mu ,\sigma )=g\left(E_{0}\right)$ where $\mu ,\sigma \in R^{d}$. We then sample λ times from N(0,I) to obtain $\left\{z^{1},\ldots z^{\lambda }\right\}$ and transform them with:

(6) $$(\mu ,\sigma )=g\left(E_{0}\right)$$

(7) $$\left\{z^{1},\ldots z^{\lambda }\right\}\sim 𝒩(0,I)$$

(8) $$p_{0}^{i}=z^{i}\cdot \sigma +\mu \;\text{for}\;i=1,..,\lambda$$

We employ an additional KL divergence regularization term to constrain the instance-aware prompt distribution as mentioned above. The parameters of g, P and Head are optimized using the objective function:

(9) $$ℒ={ℒ}_{\text{xent}}+\beta \cdot {\text{D}}_{\text{KL}}\left(N\left(\mu ,\sigma^{2}\right)\|N(0,I)\right)$$

where ${ℒ}_{\text{xent}}$ is the conventional cross-entropy loss and ${\text{D}}_{\text{KL}}(\cdot ,\cdot )$ is the KL divergence.

### 3.3 A Unified Framework for Balanced Prompt Propagation

#### Motivations.

Existing VPT variants represent opposite extremes in handling prompt information flow across transformer layers. VPT-Shallow propagates all prompt features from previous layers but lacks layer-specific adaptability, while VPT-Deep discards all previous information and introduces entirely new prompts at each layer, increasing parameter count substantially. As shown in Fig. 1, both approaches are suboptimal: discarding all prompt outputs from previous layers potentially loses valuable instance-related information, while preserving everything leaves no room for layer-specific adaptations. This observation motivates us to develop a balanced framework that selectively retains the most informative components from previous layers while allowing for new learnable parameters.

To achieve this balance, we introduce a dimension reduction strategy based on Principal Component Analysis (PCA). Let $Z_{i-1}\in R^{p\times d}$ represent the output prompt features from layer i-1. Instead of directly propagating these features (VPT-Shallow) or completely replacing them with new prompt tokens (VPT-Deep), we apply PCA to each prompt token to reduce its dimensionality. Specifically, we define a PCA transformation $\Phi :R^{p\times d}\to R^{p\times m}$ where m<d is the reduced dimension of each prompt token:

(10) $$Z_{i-1}^{\text{PCA}}=\Phi \left(Z_{i-1}\right)\in R^{p\times m}$$

Since the transformer requires input prompts of dimension d, we supplement each reduced prompt token with (d-m) learnable parameters. For each transformer layer i>1, we construct the input prompt tokens by concatenating the PCA-reduced components with learnable parameters along the feature dimension:

(11) $$P_{i-1}^{\text{combined}}=\left[Z_{i-1}^{\text{PCA}},P_{i-1}^{\text{new}}\right]\in R^{p\times d}$$

where $P_{i-1}^{\text{new}}\in R^{p\times (d-m)}$ represents randomly initialized, learnable parameters. The color ■ indicates combined parameters. In other words, each prompt token in $P_{i-1}^{\text{combined}}$ consists of two parts: the output of PCA in the first m dimensions and continuously learnable parameters in the remaining d-m dimensions. This combined prompt is then fed into the i-th transformer layer:

(12) $$\left[x_{i},Z_{i},E_{i}\right]=L_{i}\left(\left[x_{i-1},P_{i-1}^{\text{combined}},E_{i-1}\right]\right)$$

Our approach provides a continuous spectrum between VPT-Shallow and VPT-Deep through the parameter m, which controls how much information from previous layers is preserved within each prompt token. When m=0, our method reduces to VPT-Deep, as no information is carried forward from previous layers and all prompt dimensions are newly learned at each layer. Conversely, when m=d, our method becomes equivalent to VPT-Shallow, as all dimensions of prompt tokens from the previous layer are preserved with no new learnable parameters.

By setting 0<m<d, we achieve a balanced trade-off that leverages both the global context captured in earlier layers and the layer-specific adaptations. Unlike random dimension reduction, which may discard important features arbitrarily, PCA ensures that we retain the dimensions with the highest variance, effectively capturing the most informative aspects of the prompt representations. This controlled information flow allows ViaPT to maintain parameter efficiency while approaching or exceeding the performance of more parameter-intensive approaches.

The optimal value of m depends on the specific task and dataset characteristics, but our empirical results (Section ??) show that a moderate reduction (typically m≈128) consistently yields the best performance across diverse benchmarks. This supports our hypothesis that both preserving key information from earlier layers and allowing for layer-specific adaptations are complementary and essential for effective visual prompt tuning.

### 3.4 Inference Strategies and Stability

After training our ViaPT model, the inference process presents unique considerations due to the probabilistic nature of the instance-aware prompt generation. The standard inference procedure follows the same pipeline as training.

However, sampling from a Gaussian distribution introduces randomness that can lead to variations in prediction results for the same input across different inference runs. To address this challenge, we investigate several strategies to achieve stable and reliable predictions during inference.

#### Multi-round inference.

Our primary approach involves performing multiple rounds of inference for each input image and aggregating the results. Specifically, for an input image, we conduct R independent forward passes, each with a different set of randomly sampled prompt tokens. The final prediction is obtained by averaging the class probabilities across all rounds: $p_{\text{final}}=\frac{1}{R}\sum_{r=1}^{R}p_{r}$ where $p_{r}$ represents the class probability distribution from the r-th round. In our experiments, we found that R=5 provides a good balance between inference stability and computational efficiency, with the standard deviation of accuracy dropping below 0.1% across different runs.

#### Deterministic alternatives.

While multi-round inference effectively mitigates the randomness issue, there are scenarios where deterministic predictions are preferred, especially in latency-sensitive applications. We explore two alternatives to achieve deterministic inference:

##### 1. Direct prompt generation:

Instead of generating distributional parameters (μ,σ), we can modify the generator g to directly output λ prompt tokens: $g^{\prime }:R^{k\times d}\to R^{\lambda \times d}$. This approach eliminates the sampling step altogether, ensuring deterministic predictions at the cost of increased parameter count. Our experiments show that this approach achieves comparable accuracy to the probabilistic version while providing consistent predictions.

##### 2. Fixed sampling:

After training the model with sampling, we can generate a set of fixed random samples $\left\{z^{1},\ldots z^{\lambda }\right\}$ from N(0,I) and use these same samples for all images during inference. This maintains the benefits of reduced parameters while ensuring deterministic predictions. Surprisingly, this simple approach yields stable performance comparable to multi-round inference, suggesting that the learned (μ,σ) capture most of the instance-specific information needed for accurate classification.

While both deterministic alternatives offer practical advantages for deployment, we find that the original multi-round inference provides the best overall performance across our benchmarks. This suggests that the stochasticity introduced by sampling may actually benefit the model by providing a form of test-time ensemble, similar to the benefits observed in dropout during inference. The choice among these strategies ultimately depends on the specific application requirements, with trade-offs between accuracy, stability, and computational efficiency.

## 4 Experiments

### 4.1 Experiment Setup

#### Datasets.

We conduct extensive evaluations on three widely adopted image classification benchmarks: **HTA** [26], **VTAB-1k** [70], and **FGVC** [55]. HTA is a recent benchmark to evaluate prompt tuning methods under diverse task and domain shifts. It comprises ten classification datasets that vary in granularity, modality, and semantic complexity, including CIFAR-10, CIFAR-100 [32], DTD [12], CUB-200 [56], NABirds [54], Oxford Flowers [42], Food101 [6], GTSRB [51], and SVHN [41]. We follow the official experimental settings provided in DAM-VP [26] to ensure direct comparability with previous works. VTAB-1k consists of 19 diverse tasks categorized into three groups: (1) *Natural*, involving real-world images captured by standard cameras; (2) *Specialized*, featuring data from domain-specific devices (e.g., satellites, medical scanners); and (3) *Structured*, focusing on geometric reasoning and synthetic scenes (e.g., object counting, position encoding). The structured tasks are semantically distant from the ImageNet-21k [14] pretraining distribution, making them more challenging. Each VTAB task includes 1,000 training examples with a standard 800/200 train/val split. The FGVC benchmark covers 5 fine-grained classification datasets: CUB-200-2011, NABirds, Oxford Flowers, Stanford Dogs, and Stanford Cars, where each dataset is split into 90% train and 10% val.

#### Baselines.

We benchmark ViaPT against a variety of PEFT methods as outlined by [68]. All comparisons utilize ViT-Base/16 [16] backbone pretrained on ImageNet-21k to ensure fairness and reproducibility. Detailed comparisons including both supervised and self-supervised pretrained variants, such as MAE [22] and MoCo v3 [10], are discussed in §4.4.

### 4.2 Comparison with State-of-the-Art Methods

As shown in Table 1, our method consistently achieves the best performance across all evaluated benchmarks. Specifically, we obtain the highest average accuracy on **FGVC (91.40%)**, **HTA (92.20%)**, and **VTAB-1k (76.36%)**, outperforming not only full fine-tuning (88.54%, 85.80%, 65.57%) but also strong prompting baselines such as SA^2^VP [44] and VFPT [68]. Particularly on *Structured* tasks in VTAB-1k, which are known to exhibit significant distributional shift, our method achieves 61.25%, surpassing SA^2^VP by +0.45%. It is worth noting that although we retain the same number of prompt tokens as standard VPT, we introduce a semantic bottleneck within each token by projecting into a task-relevant subspace via PCA. This design reorganizes each prompt token’s representation rather than compressing the total token count, effectively filtering out redundant directions while preserving the most informative semantic axes. Compared to VPT-D [29], which uses a universal prompt shared across all samples, our instance-aware mechanism dynamically generates personalized prompts for each input, significantly improving performance on datasets with large intra-class variation, such as CUB-200, Pets, and DTD. To further disentangle the impact of each component, we provide an ablation study in Table 4, clearly showing that both instance-aware prompting and PCA projection in our method are essential to the overall improvement. Despite operating under a strict parameter budget (**0.74%** trainable parameters), our approach establishes new state-of-the-art results across all settings. Compared to SA^2^VP [44], which adopts a similar tuning ratio (0.76%) but falls short by **0.85%** on average, ours achieves better adaptation by jointly leveraging prompt subspace modeling and instance-level semantics within a unified framework. In addition, an interesting observation on VTAB-1k is that VPT related methods including ours show better results when source and target domains have a relatively big gap, well aligned with the finding in [20].

#### Parameter efficiency.

As shown in Table 1, our method has fewer learnable parameters than most of the competitors. Notably, while VPT-S (shallow) holds much fewer parameters, it yields much lower performance since randomized learnable parameters are only applied at the input layer. E2VPT focuses on distillation, thus showing an advantage of parameter amount.

### 4.3 Generalization to Other Backbones

To assess the generality of our method across architectures, we evaluate its performance using the Swin-Base [38] backbone pretrained on ImageNet-21k. Table 3 presents the results on VTAB-1k, comparing our approach with both classical and recent parameter-efficient tuning methods.

#### Consistent improvements across all VTAB categories.

Our method achieves the highest accuracy in the *Natural* (85.29%) and *Structured* (62.24%) categories, while matching the best in *Specialized* (86.21%). In particular, our structured-task performance surpasses the strongest baseline (SA^2^VP) by +2.21%, suggesting that our method is better at capturing spatial dependencies and relational patterns that are prevalent in counting and geometric reasoning tasks.

#### Improved robustness under architectural shift.

Although most visual prompting methods are initially developed for standard ViT architectures, our method retains strong performance when applied to hierarchical Transformer-based backbones such as Swin. This demonstrates that our semantic compression and instance-aware prompting mechanisms are not tightly coupled to a specific ViT formulation, and can effectively generalize across a variety of Transformer-based vision models.

### 4.4 Effectiveness Across Pretraining Paradigms

To evaluate the robustness and generality of our approach under different pretraining paradigms, we compare the methods using ViT-Base/16 models pretrained with either MAE [22] or MoCo v3 [10]. As shown in Table 2, our method shows competitive performance with a moderate parameter overhead, demonstrating its strong compatibility with different pretraining schemes.

**Under MAE pretraining,** our method achieves competitive performance, notably outperforming prior parameter-efficient baselines such as VPT [29], GPT [62], and VFPT in the *Specialized* category (+0.26% over VFPT), which often includes domain-specific tasks (e.g., medical, satellite images). Despite MAE’s inherent information loss due to reconstruction-based learning, our approach is able to extract and refine meaningful representations through instance-aware prompt sampling.

**Under MoCo v3 pretraining,** our method consistently surpasses all baselines, achieving top results in all three VTAB-1k categories. In particular, we observe notable gains in the *Structured* group (+1.31% over VFPT), which tends to exhibit distributional shifts far from the pretraining domain. These results reveal that the instance-aware latent space introduced by our prompt encoder provides enhanced resilience to domain gaps, even under contrastive pretraining that prioritizes instance-level discrimination.

#### Prompt robustness to pretraining variance.

Prior studies showed that prompt-based tuning may suffer from unstable performance under self-supervised pretraining [67, 75]. In contrast, our method yields *consistent gains across all categories and paradigms*, suggesting that subspace-guided prompt decomposition improves the semantic reliability of learned tokens, regardless of upstream objective.

### 4.5 Ablation Study

To understand the contribution of each core component in our framework, we conduct a comprehensive ablation study on the VTAB-1k benchmark using ViT-Base/16 pretrained on ImageNet-21k. As shown in Table 4, we systematically remove or replace key modules to evaluate their individual and combined effects.

#### Effectiveness of instance-aware prompts.

When removing the instance-aware prompting while retaining PCA compression, the average VTAB-1k accuracy drops from 76.36% to 75.03%. This 1.33% degradation confirms that static dataset-level prompts are suboptimal for handling input variability, and highlights the benefit of injecting personalized prompt information on a per-instance basis.

#### Trade-off between efficiency and accuracy.

To further explore the efficiency of our prompt strategy, we visualize the ratio of trainable parameters to the total model size across varying prompt dimensionalities in Fig. 3. The instance-aware variant (blue) maintains a consistently lower parameter ratio compared to the dataset-level prompt baseline (pink), particularly when the dimensionality is reduced. This reflects the parameter advantage of our design, which offloads representation burden from prompt tokens to the lightweight instance generator. Notably, despite tuning fewer parameters, our method achieves superior or comparable accuracy at nearly every dimension (Fig. 1). These results suggest that semantic compression not only improves performance but also yields better parameter efficiency, offering a favorable trade-off in resource-limited scenarios.

#### Benefit of semantic compression via PCA.

As shown in Fig 1, eliminating the PCA compression while keeping instance-aware prompts results in a 2.01% drop in average accuracy (from 76.36% to 74.35%). This gap demonstrates the importance of semantic bottlenecking: PCA selects task-relevant latent directions, filters noisy or redundant features, and facilitates stable prompt transfer across transformer layers.

#### Complementarity between components.

The most substantial performance drop (3.18%) occurs when both components are disabled (i.e., dataset-level random prompts without PCA), resulting in 73.18% accuracy. This indicates that PCA and instance prompts not only contribute individually but also reinforce each other: semantic compression guides prompt generation towards informative subspaces, while instance conditioning ensures adaptiveness within that subspace.

### 4.6 Interpretability and Feature Representation

To gain deeper insights into how our instance-aware and PCA-refined prompts influence the learned representations, we conduct qualitative and quantitative interpretability analyses, including Grad-CAM visualizations, t-SNE embeddings, and token similarity heatmaps. These tools help uncover the semantic structure, instance adaptivity, and representational compactness of the proposed method.

#### Heatmap Visualization via Grad-CAM.

To explore how prompt design influences attention allocation, we visualize class-specific activation heatmaps using Grad-CAM [49] over the [CLS] token in the final transformer layer. As shown in Fig. 4, VPT-Deep often exhibits diffused or off-target activation, failing to focus on semantically relevant regions. In contrast, our instance-aware prompt generation produces sharper and more centralized heatmaps, consistently aligning with the object of interest (e.g., the bird body or head), indicating improved feature localization and interpretability.

#### t-SNE Embedding Analysis.

To assess the effectiveness of our instance-aware prompt learning from a representational perspective, we visualize the output class tokens $x_{N}$ using t-SNE [53]. Fig. 5 presents a comparative visualization of the embeddings produced by VPT-Deep and our ViaPT on the CUB-200 dataset with five randomly sampled categories. The embeddings derived from our method exhibit markedly improved cluster compactness and inter-class separability, forming clearly delineated and semantically structured regions in the latent space. This suggests that instance-specific prompt modeling contributes to more consistent intra-class alignment and stronger discriminative capability. In contrast, VPT-Deep embeddings show significant overlap between classes, indicating weaker category specificity and feature confusion. These results corroborate the hypothesis that our proposed framework facilitates superior semantic organization in the feature space.

#### Prompt Token Similarity Visualization.

To further understand the effectiveness of our instance-aware prompt design, we visualize the prompt token similarity maps for selected images. Specifically, we compute cosine similarity between prompt tokens and spatial locations at the first transformer layer. As illustrated in Fig. 6, VPT-Deep produces highly sparse and noisy activations, with attention scattered across background regions. In contrast, our method exhibits more concentrated and semantically meaningful attention patterns—focusing on task-relevant parts such as the dog’s face and the vase’s spout. This suggests that our instance-aware prompt generation effectively guides attention to critical visual cues, leading to improved prompt utility and reduced redundancy.

## 5 Conclusion

We reveal that instance-aware prompts can significantly boost prompt tuning for vision transformers. This is achieved by the proposed ViaPT method. We show that the key to ViaPT includes generating instance-aware prompts based on each individual input and leveraging PCA to retain important prompting information when fusing dataset-level and instance-aware prompts. Based our proposed conceptual understanding, the relationship between ViaPT and typical VPT schemes is interpreted, indicating the rationale of our method from another perspective. The promising results on diverse and challenging experiments demonstrate the competitiveness of ViaPT, potentially benefiting the research communities of deep transfer learning and foundation models.

## Figures and Tables

**Figure 1:**
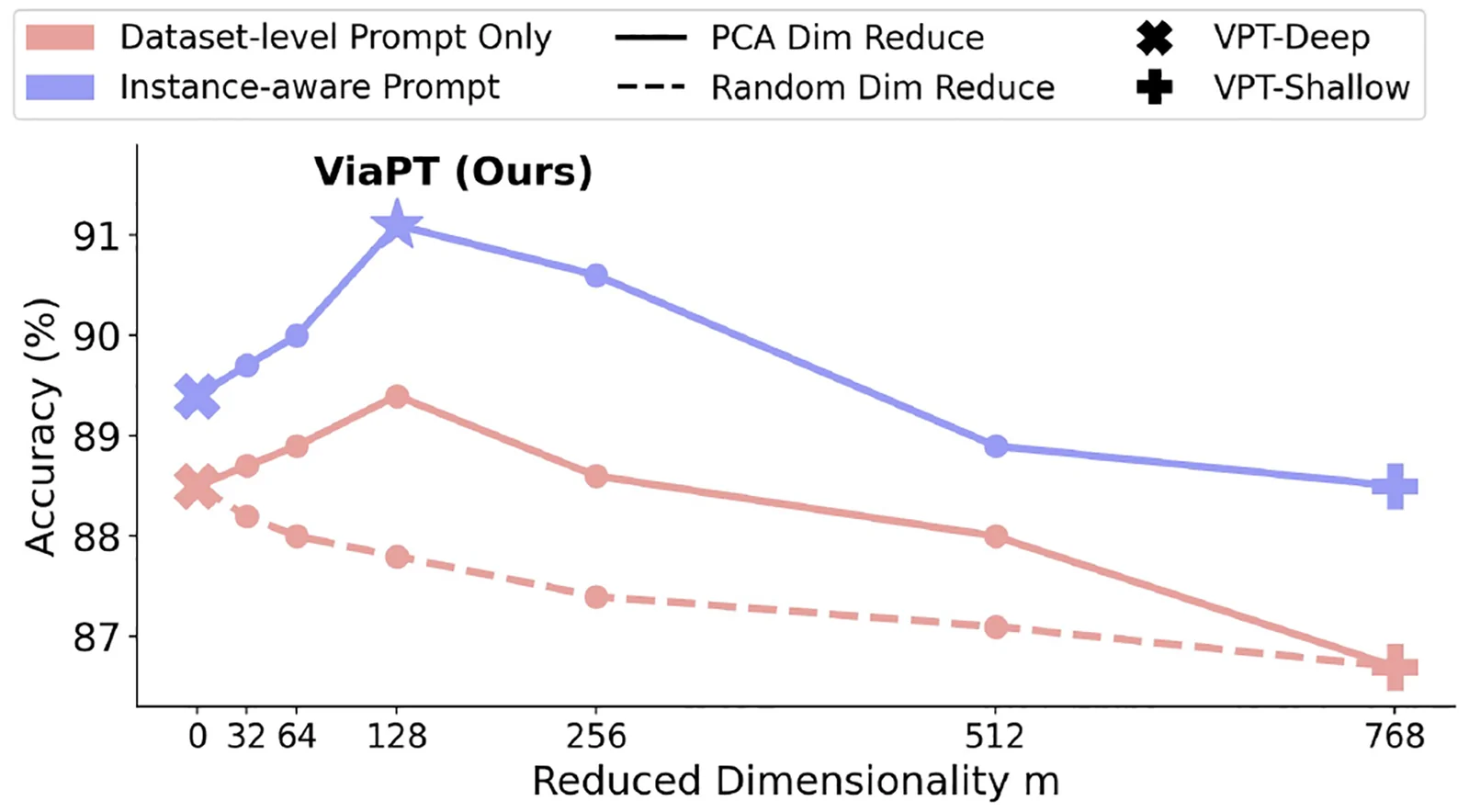
Balancing information flow and instance awareness for optimal adaptation. Our framework (pink) reveals VPT-Shallow and VPT-Deep as corner cases (m=768 and m=0), demonstrating the importance of controlled information flow across layers. Combining with instance-aware prompts (blue) further improves performance across all configurations, with peak accuracy at a medium dimensionality (m=128) highlighting the dual benefit of instance awareness and balanced information propagation.

**Figure 2:**
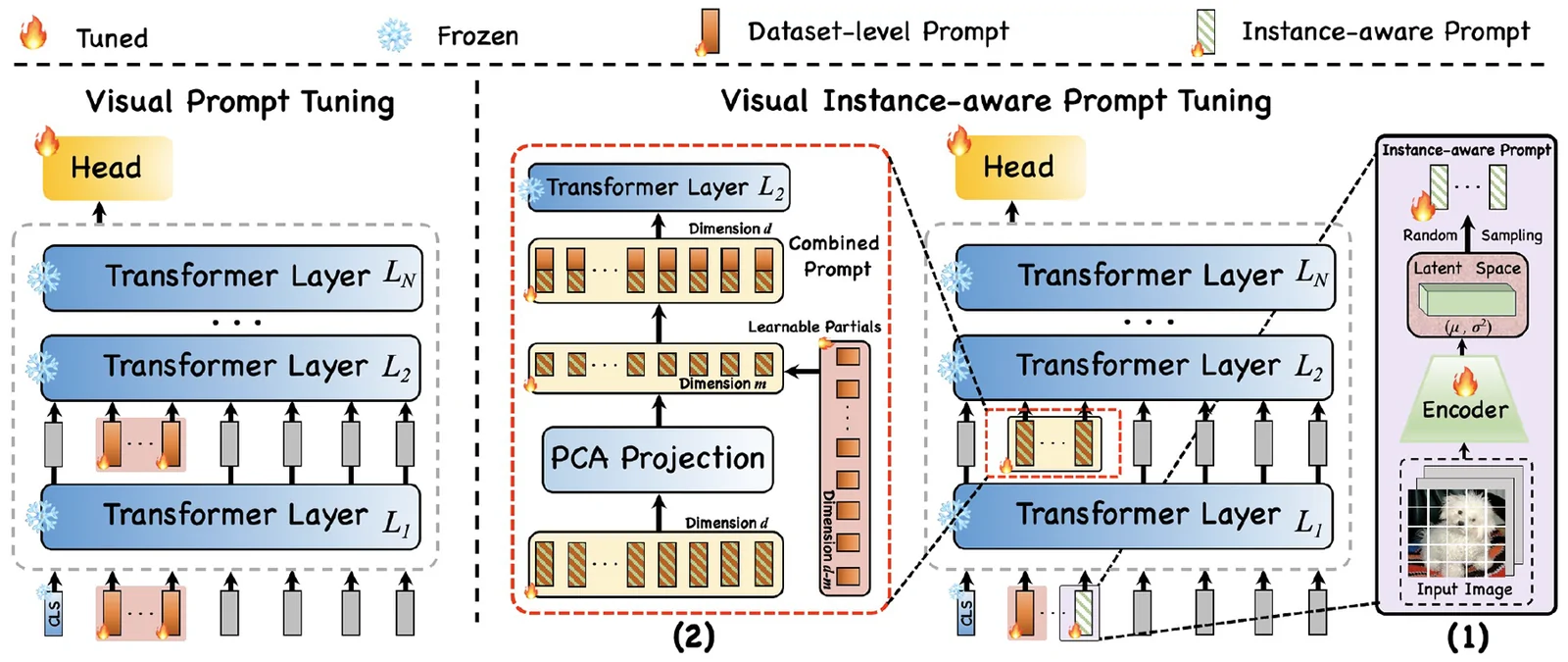
Overview of the proposed ViaPT framework. Left: Conventional VPT uses dataset-level learnable prompts that remain identical across all inputs. Right: Our ViaPT introduces: (1) an Instance-aware Prompt Generator that creates input-specific prompts through a minimal encoder using the reparameterization trick [31]; (2) a Balanced Prompt Propagation module that combines PCA-projected outputs (to retain high-variance components in dimension m) from previous layers with learnable parameters (dimension d-m) to optimize information flow across transformer layers.

**Figure 3:**
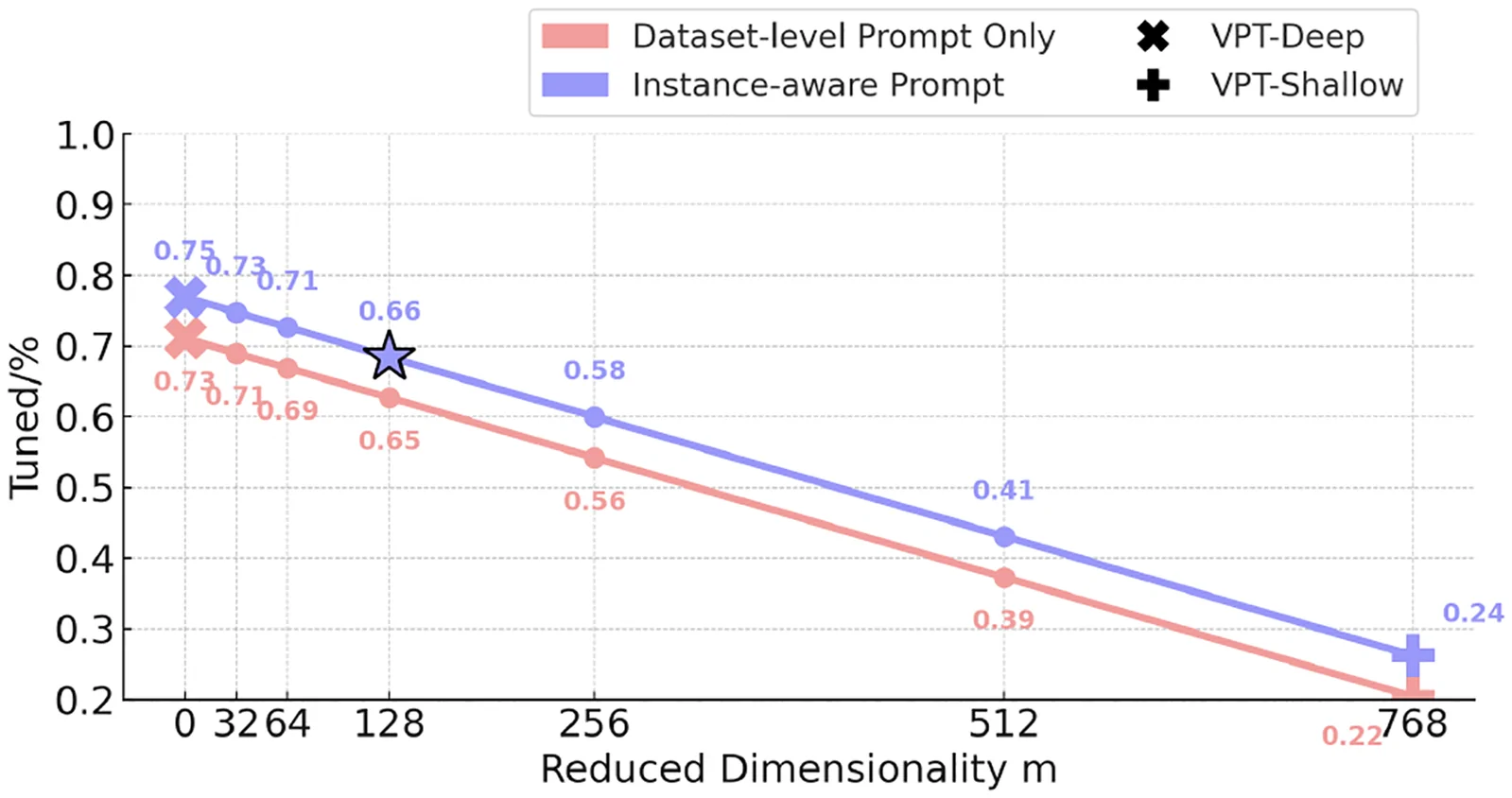
Parameter efficiency vs. dimensionality. Trainable/total parameter ratio across dimensionalities (m).

**Figure 4:**
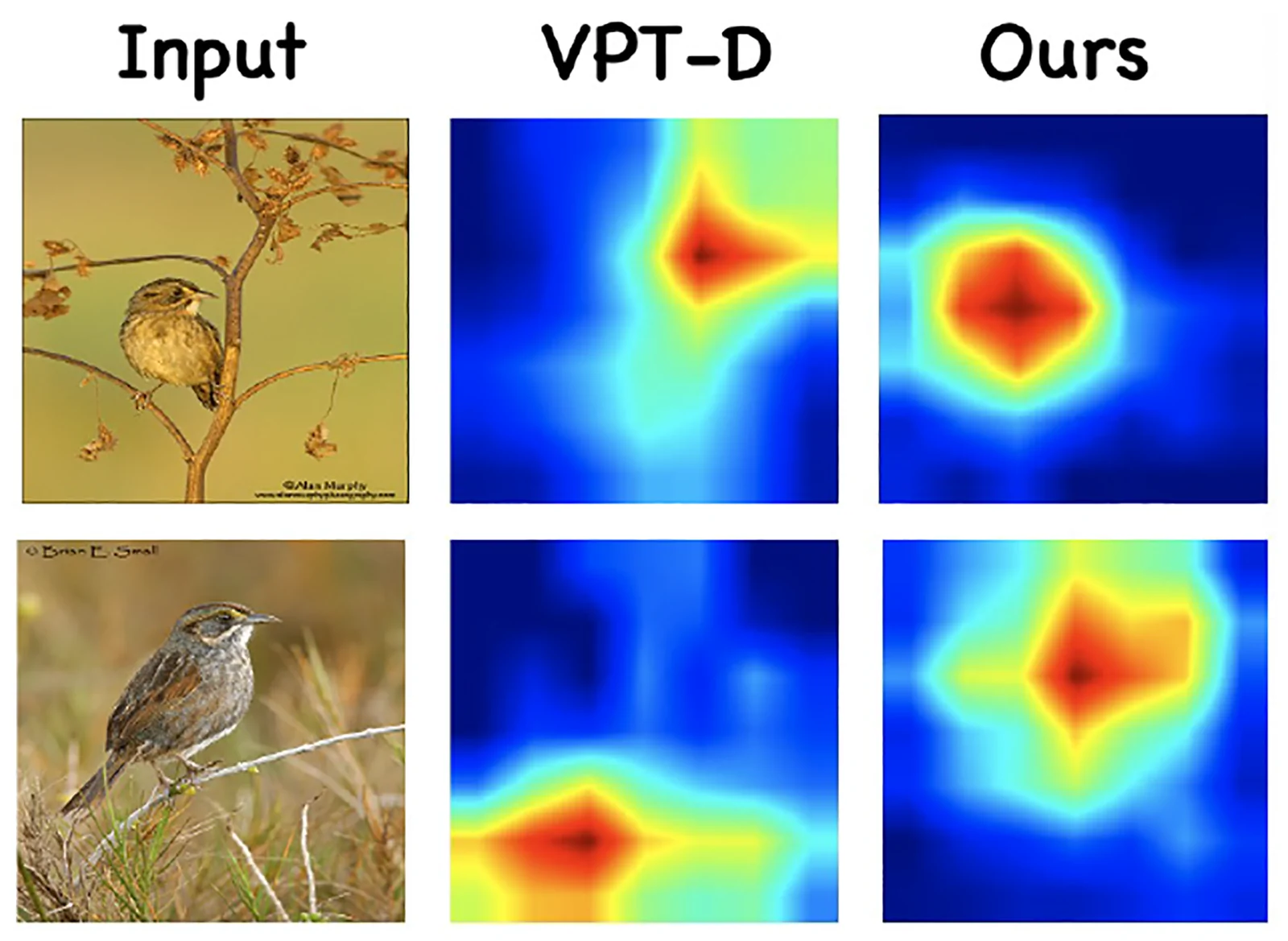
Grad-CAM heatmaps on CUB-200 samples. Compared to VPT-Deep, ViaPT generates more focused and semantically aligned heatmaps, demonstrating better spatial consistency with object regions.

**Figure 5:**
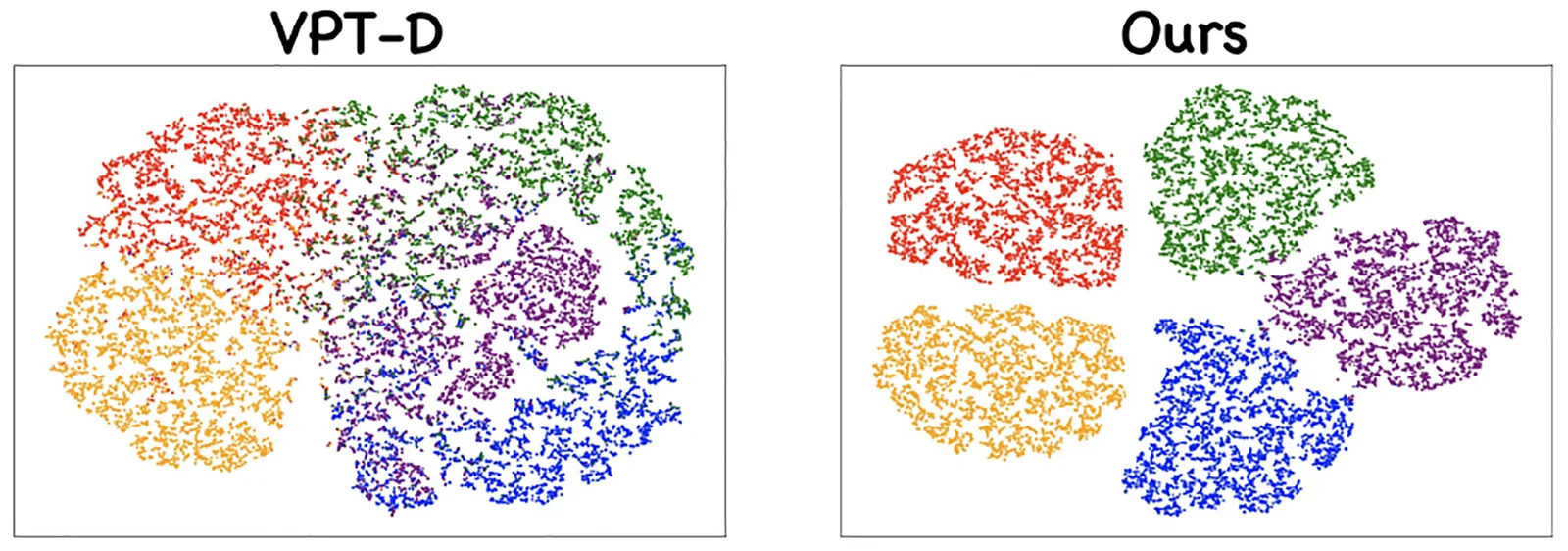
t-SNE visualization on CUB-200. The proposed ViaPT yields compact and well-separated clusters across five categories, demonstrating enhanced semantic discriminability compared to VPT-Deep.

**Figure 6:**
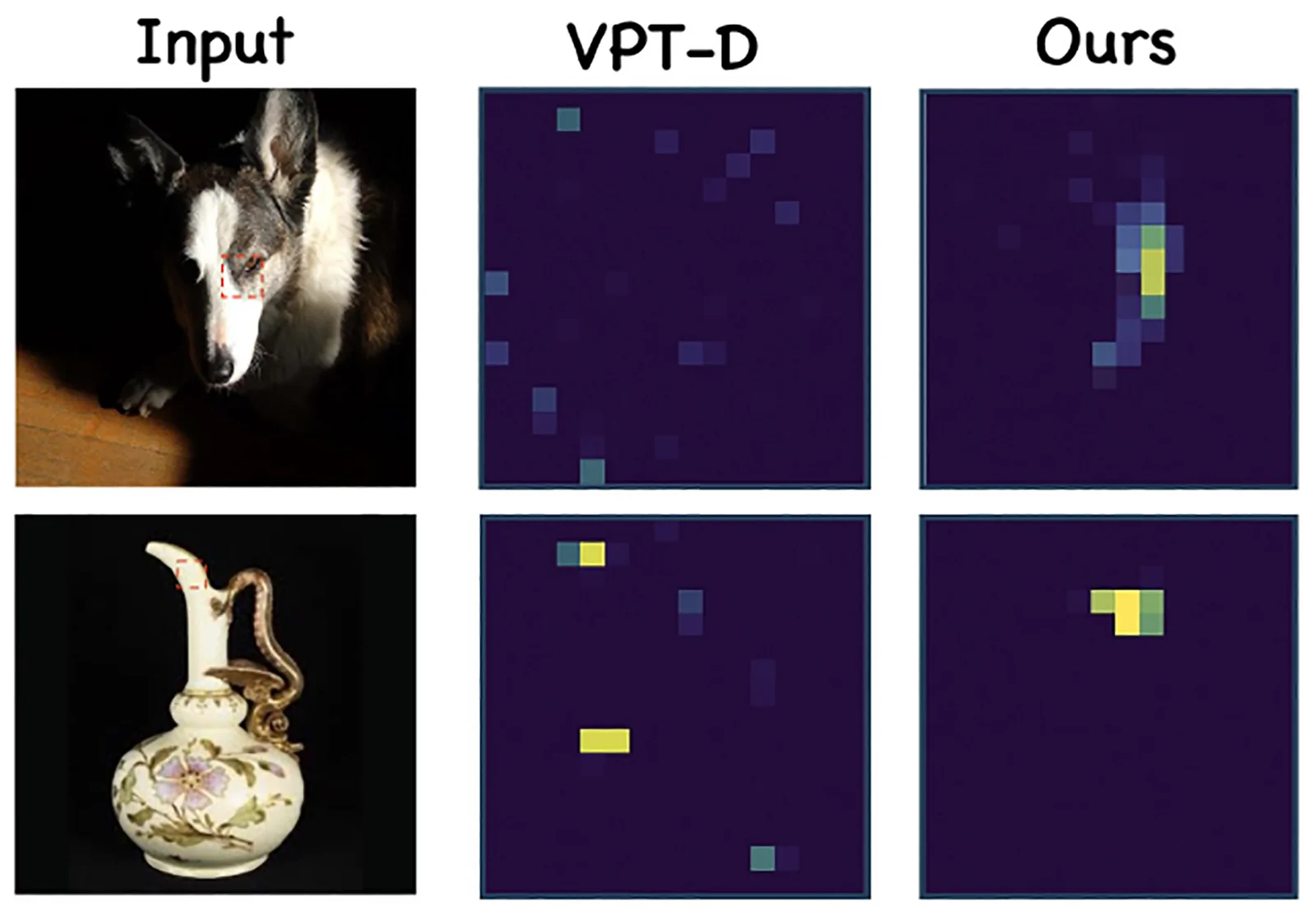
Prompt token similarity heatmaps. Compared with VPT-Deep, our instance-aware prompts yield more focused and semantically meaningful attention maps.

**Table 1:** Comparison of fine-tuning methods with ViT-Base/16 backbone. Bold and underlined indicate best and second-best results. We report mean accuracy across datasets for each benchmark (FGVC: 5 datasets, HTA: 10 datasets, VTAB-1k: 19 datasets) and for each VTAB-1k category (Natural, Specialized, and Structured). See Appendix ?? for per-task results. Same for Table 2.

ViT-Base/16 [16] (85.8M)	Tuned/Total (%)	Extra Params	FGVC Mean (%)	HTA Mean (%)	VTAB-1k [70]			VTAB-1k Mean (%)
					*Natural*	*Specialized*	*Structured*	
Full [CVPR22][27]	100.00	–	88.54	85.8	75.88	83.36	47.64	65.57
Linear [CVPR22][27]	0.08	–	79.32	75.7	68.93	77.16	26.84	52.94
Partial-1 [NeurIPS14][63]	8.34	–	82.63	80.8	69.44	78.53	34.17	56.52
MLP-3 [CVPR20][9]	1.44	✓	79.80	78.5	67.80	72.83	30.62	53.21
Sidetune [ECCV20][71]	10.08	–	78.35	72.3	58.21	68.12	23.41	45.65
Bias [NeurIPS17][46]	0.80	–	88.41	82.1	73.30	78.25	44.09	62.05
Adapter [NeurIPS20][7]	1.02	✓	85.46	80.6	70.67	77.80	33.09	62.41
LoRA [ICLR22][24]	–	✓	89.46	85.5	78.26	83.78	56.20	72.25
AdaptFormer [NeurIPS22][8]	–	✓	89.12	89.0	80.56	84.88	58.38	72.32
ARC_att_ [NeurIPS23][15]	–	✓	89.12	89.0	80.41	85.55	58.38	72.32
VPT-S [ECCV22][29]	0.16	✓	84.62	85.5	76.81	79.66	46.98	64.85
VPT-D [ECCV22][29]	0.73	✓	89.11	85.5	78.48	82.43	54.98	69.43
E2VPT [ICCV23][19]	0.39	✓	89.22	88.5	80.01	84.43	57.39	71.42
EXPRES [CVPR23][13]	–	✓	–	–	79.69	84.03	54.99	70.02
DAM-VP [CVPR23][26]	–	✓	–	88.50	–	–	–	–
SA^2^VP [AAAI24][44]	0.76	✓	90.08	91.50	80.97	**85.73**	60.80	75.83
VFPT [NeurIPS24][68]	0.66	✓	89.24	–	81.35	84.93	60.19	75.49
LoR-VP [ICLR25][30]	–	✓	89.32	–	79.91	83.16	60.01	74.36
**Ours**	0.66	✓	**91.40**	**92.20**	**82.62**	85.22	**61.25**	**76.36**

**Table 2:** Comparison of the fine-tuning methods under different pretraining paradigms. We report VTAB-1k [70] accuracy using ViT-Base [16] as the frozen backbone, pretrained with MAE [22] and MoCo v3 [10] respectively. Our method shows competitive performance with a moderate parameter overhead.

Pretraining	MAE [22]				MoCo v3 [10]			
Method	Tuned/%	Natural	Specialized	Structured	Tuned/%	Natural	Specialized	Structured
Full [CVPR22][27]	100.00%	59.31%	79.68%	53.82%	100.00%	71.95%	84.72%	51.98%
Linear [CVPR22][27]	0.04%	18.87%	53.72%	23.70%	0.04%	67.46%	81.08%	30.33%
Partial-1 [NeurIPS14][63]	8.30%	**58.44%**	**78.28%**	47.64%	8.30%	72.31%	84.58%	47.89%
Bias [NeurIPS17][46]	0.16%	54.55%	75.68%	**47.70%**	0.16%	72.89%	81.14%	53.43%
Adapter [NeurIPS20][7]	0.87%	54.90%	75.19%	38.98%	1.12%	74.19%	82.66%	47.69%
VPT-S [ECCV22][29]	0.05%	39.96%	69.65%	27.50%	0.06%	67.34%	82.26%	37.55%
VPT-D [ECCV22][29]	0.31%	36.02%	60.61%	26.57%	0.22%	70.27%	83.04%	42.38%
GPT [arXiv24][62]	0.05%	47.61%	76.86%	36.80%	0.06%	74.84%	83.38%	49.10%
VFPT [NeurIPS24][68]	0.38%	53.59%	77.75%	36.15%	0.22%	77.47%	85.76%	58.74%
**Ours**	0.36%	54.26%	78.01%	37.52%	0.30%	**79.12%**	**86.81%**	**60.05%**

**Table 3:** Method comparison on VTAB-1k using Swin-Base model pretrained on ImageNet-21k.

Swin-Base (86.7M)	Tuned/Total (%)	VTAB-1k		
		*Natural*	*Specialized*	*Structured*
Full [CVPR22][27]	100.00	79.10	86.21	59.65
Linear [CVPR22][27]	0.06	73.52	80.77	33.52
Bias [NeurIPS17][46]	0.30	76.78	83.33	51.85
VPT-deep [ECCV22][29]	0.25	76.78	83.33	51.85
E^2^VPT [ICCV23][19]	0.21	83.31	84.95	57.35
SA^2^VP [AAAI24][44]	0.29	80.81	**86.30**	60.03
VFPT [NeurIPS24][68]	0.27	84.53	86.15	58.21
**Ours**	0.27	**85.29**	86.21	**62.24**

**Table 4:** Ablation study on instance-aware prompt and PCA-based decomposition using ViT-Base/16 on VTAB-1k.

Method	Natural	Specialized	Structured	VTAB-1k Mean
w/o PCA	80.72	83.14	59.18	74.35
w/o Instance Prompt	81.15	83.90	60.05	75.03
w/o PCA & Inst.	79.68	81.94	57.92	73.18
w/Random Prompt	80.10	82.53	58.70	73.78
**Full Model (Ours)**	**82.62**	**85.22**	**61.25**	**76.36**

## CCS Concepts

- **Computing methodologies → Artificial intelligence**; *Computer vision representations*.
